# NARRATIVE AS A BOUNDARY CONCEPT IN GERONTOLOGY: THE STORY BEHIND THE STORIES

**DOI:** 10.1093/geroni/igac059.959

**Published:** 2022-12-20

**Authors:** Kate de Medeiros, Desmond O'Neill

## Abstract

Ever since the “narrative turn” was taken up by the humanities and social sciences in the 1980s and early 1990s, there has been an increased interest in narrative as a medium for gaining knowledge in gerontology. Narrative gerontology in particular assumes that the "life lived is inseparable from the life told" (Bruner 1987). Central to this notion is the metaphor of life as story or, as Kenyon and Randall put it, "[W]e not only have stories, we are stories" (1999). As a boundary concept, “narrative” inhabits several disciplinary worlds. Its analysis can focus on individual identification strategies, on the storytelling process and its context, or on aesthetic dimensions, for instance when analyzing cultural representations including film and fiction. Often loosely defined yet broadly applied, narrative work can range from personal life stories to master narratives that convey cultural and/or political values. The purpose of the Humanities and Arts symposium is to take a critical look at "narrative" within gerontology to include mapping the uses of narrative as actions, objects, ways of knowing and acts of resistance. The first paper presents a conceptual structure of narrative based on findings from a guided review of narrative definitions and approaches in gerontology. The second argues for the normative importance in studying cultural narratives of dementia. The third paper examines narrative in the context of the medical humanities and narrative medicine, pointing to ways in which narrative competence translates into medical practice. The final paper considers the narrative of aging through music.

